# A Rare Case of Right-Sided Aortic Arch With Mirror-Image Branching and Congenital Absence of the Left Pulmonary Artery

**DOI:** 10.7759/cureus.38242

**Published:** 2023-04-28

**Authors:** Jurgen Shtembari, Dhan B Shrestha, Kaiyuan Zhang, Wasey Ali Yadullahi Mir

**Affiliations:** 1 Department of Internal Medicine, Mount Sinai Hospital, Chicago, USA; 2 Department of Internal Medicine, Ross University School of Medicine, Bridgetown, BRB

**Keywords:** aortic arch, congenital defect, malformation, thoracic aorta, pulmonary artery

## Abstract

Both the right-sided aortic arch with mirror-image branching (RAMI) and the congenital absence of the left pulmonary artery are sporadic congenital defects. Both diseases are typically diagnosed in childhood, but occasionally asymptomatic cases may be incidentally detected through imaging in adulthood. We reported a 43-year-old female patient with a RAMI and congenital absence of the left pulmonary artery who was relatively asymptomatic until adulthood.

## Introduction

Unilateral absence of the pulmonary artery (UAPA) results from the involution of the proximal sixth aortic arch, with an observed rate of ~ 1/200,000 [1]. The congenital absence of the left pulmonary artery is less frequent than the absence of the right pulmonary artery [2]. However, left-sided agenesis is often accompanied by concurrent congenital cardiovascular malformation [3].

On the other hand, the right aortic arch with mirror-image branching (RAMI) in the general population usually remains asymptomatic and undetected; there is a 0.012-0.018% prevalence of RAMI detected in adult CT examinations [4]. RAMI results from the regression of the left dorsal aorta distal to the origin of the intersegmental artery; therefore, the left fourth arch transforms into the proximal subclavian artery instead of the definitive aortic arch [5].

## Case presentation

Our patient is a 43-year-old female who was evaluated in our hospital for dyspnea on exertion. She reported shortness of breath after walking two blocks or climbing one flight of stairs. She also endorsed nocturnal orthopnea, requiring three pillows during sleep, atypical left-sided chest pain, and daily palpitations.

Initial physical examination revealed left-shifted trachea and left chest hypokinesia on inspection, reduced breath sounds over the left hemithorax on auscultation, and left-sided dullness on percussion.

**Figure 1:**
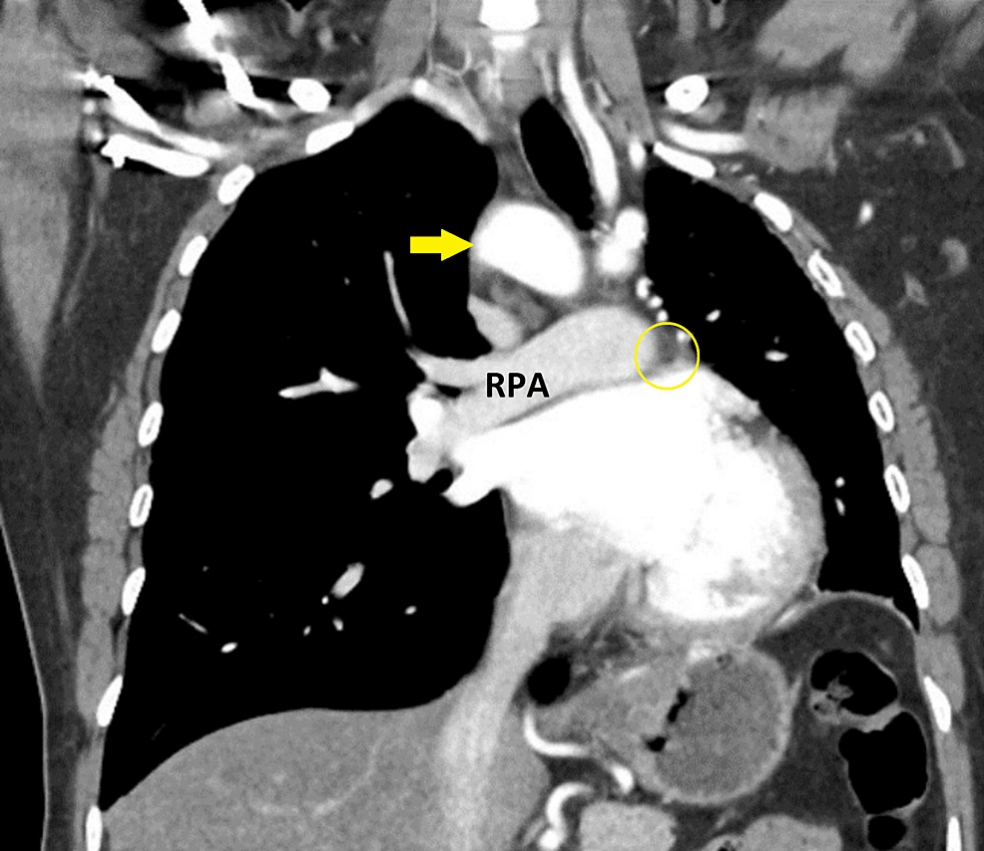
RAMI (yellow arrow) and missing left pulmonary artery (yellow circle) RAMI: right-sided aortic arch with mirror-image; RPA: right pulmonary artery

**Figure 2:**
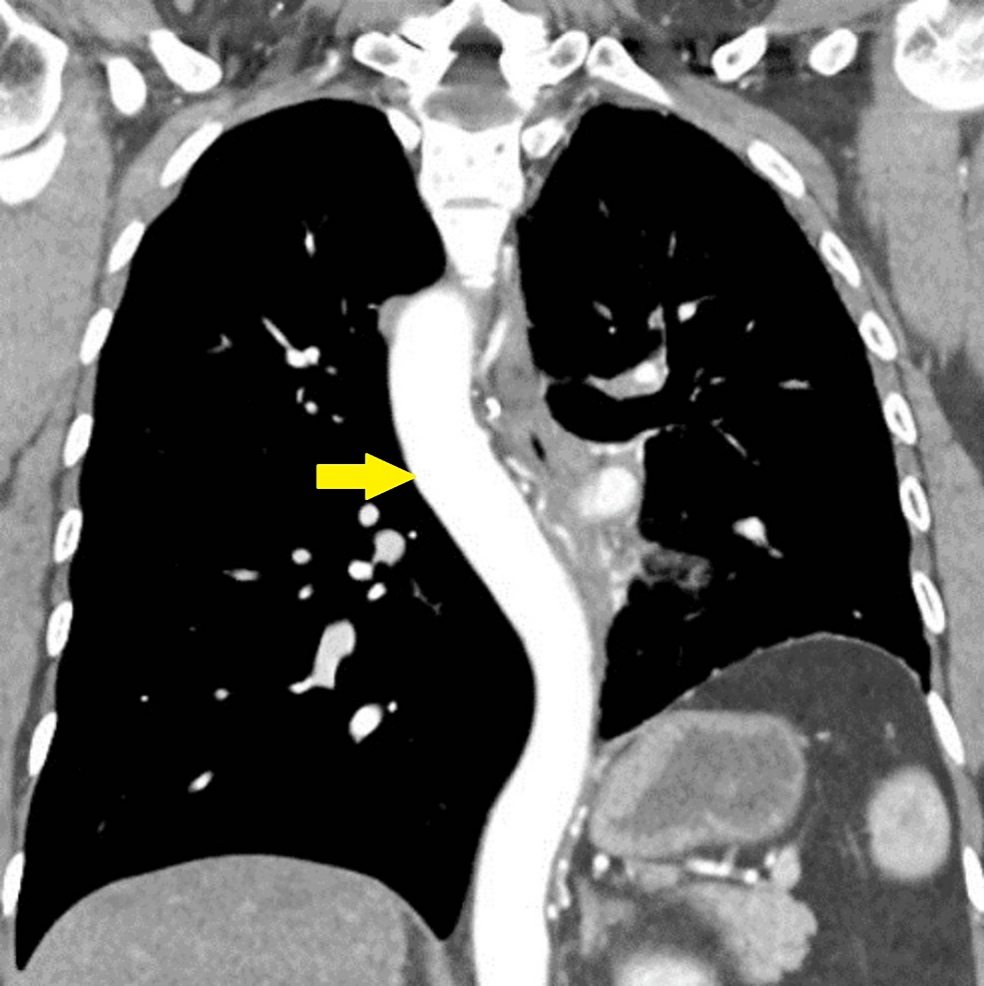
Right-sided aortic arch with mirror-image (yellow arrow)

**Figure 3:**
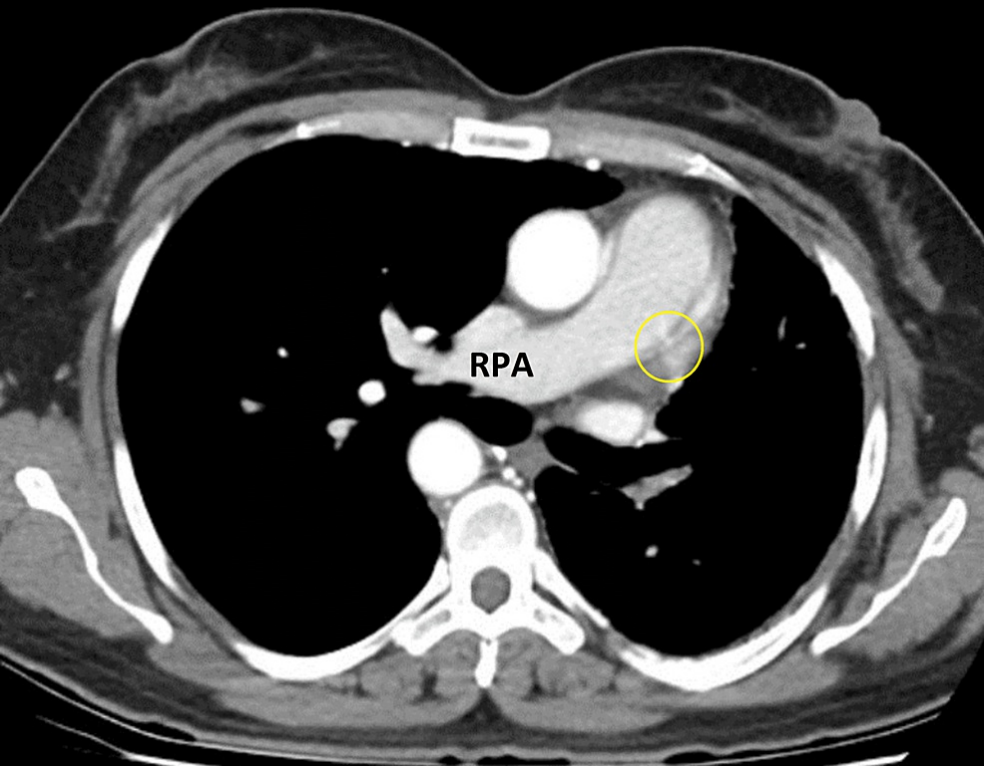
Unilateral right pulmonary artery with absence of the left pulmonary artery (yellow circle) RPA: right pulmonary artery

A chest X-ray showed a compressed left hemithorax and compensatory right lung hyperinflation. The final diagnosis was made after a chest CT with contrast showed a hypoplastic left lung with a diminutive single left pulmonary vein in addition to a right-sided aortic arch with mirror-image branching; the aorta passed posteriorly to the right main stem bronchus and crossed to the left side in the abdomen (Figures 1-3). An echocardiogram showed no cardiac abnormalities, and Holter monitoring was done to rule out rhythm disorders.

Our patient was managed conservatively with regular follow-up monitoring.

## Discussion

The unilateral absence of a pulmonary artery is a very rare congenital disease. The predominant right-sided pulmonary artery agenesis is twice as frequent as the left-sided [2]. Common associated cardiovascular comorbidities with UAPA include septal defects, right aortic arch, tetralogy of Fallot, and persistence of the ductus arteriosus [6]. Approximately 80% of the left pulmonary artery agenesis is accompanied by congenital cardiovascular abnormalities and may present with severe symptoms in early life [1]. However, our patient was not diagnosed until adulthood.

Normally, the part of the right dorsal aorta rises from the right seventh intersegmental artery till the common descending aorta disappears. However, the abnormal right-sided aortic arch like in the current case results from the persistence of the dorsal aorta on the right side and its obliteration on the left side. The persistence of this embryonic segment on both sides causes another congenital anomaly called the double aortic arch. In such a case, the double arch forms a ring that can encircle the trachea and esophagus and can lead to dyspnea and dysphagia [7].

A retrospective analysis consisting of 108 cases of UAPA concluded that the most common symptoms include pulmonary hypertension (44%), dyspnea or limited exercise tolerance (40%), frequent pulmonary infections (37%), or hemoptysis (20%) [3]. Relatively asymptomatic patients have collateral blood supply from either a patent ductus arteriosus, bronchial collaterals, other smaller aortic branches, or, less commonly, transpleural intercostal arteries to the affected side [8]. The presence of collateral lung supply could have contributed to our patient’s late onset of symptoms.

There are various treatment options for UAPA patients, and treatment selection should be patient-focused. For young patients, the goal should be to promote revascularization of the distal affected pulmonary artery. This can be achieved through a systemic-to-pulmonary artery shunt if the intrapulmonary arteries are suitable [1,3]. Pneumonectomy or selective embolization of the systemic arteries are the options for recurrent hemoptysis [6,9,10].

Pulmonary artery hypertension is a major complication in adult patients, and a case report indicated that the combination of bosentan and warfarin might be beneficial in improving symptoms [11,12]. Recurrent respiratory infections are also commonly associated with relatively asymptomatic adult patients. Physiological bronchoconstriction responses to alveolar hypocapnia and impaired mucociliary clearance are risk factors [3]. 

Our patient developed a symptomatic course of the disease in adulthood. She has no history of recurrent respiratory infections or hemoptysis. Due to her age and the nature of her symptoms, continuous follow-ups with symptomatic management were recommended.

## Conclusions

The coincidence of UAPA and RAMI is an extremely rare congenital cardiovascular anomaly. Asymptomatic patients can present later in adulthood and can be safely followed up with regular monitoring of cardiopulmonary functions.
